# Tobacco smoking negatively influences the achievement of greater than three-quarters reduction in psoriasis area and severity index after eight weeks of treatment among patients with psoriasis: Findings from a prospective study

**DOI:** 10.18332/tid/184143

**Published:** 2024-04-11

**Authors:** Yan Qiang, Le Kuai, Shuo Liu, Quanruo Xu, Lingzi Shenfan, Rui Zhang, Zhongzhi Gao, Xiangjin Gao, Bin Li, Ruiping Wang

**Affiliations:** 1 Clinical Research Center, Shanghai Skin Diseases Hospital, School of Medicine, Tongji University, Shanghai, China; 2 Department of Dermatology, Yueyang Hospital of Integrated Traditional Chinese and Western Medicine, Shanghai University of Traditional Chinese Medicine, Shanghai, China; 3 School of Public Health, Shanghai University of Traditional Chinese Medicine, Shanghai, China

**Keywords:** psoriasis, psoriasis area severity index (PASI), smoker, treatment efficacy

## Abstract

**INTRODUCTION:**

Smoking is an independent and modifiable risk factor for the onset and development of psoriasis; however, evidence on the association between tobacco smoking and psoriasis treatment efficacy is limited. This study aimed to explore the influence of smoking on treatment efficacy in a cohort of patients with psoriasis in Shanghai, China.

**METHODS:**

Patients with psoriasis were recruited from the Shanghai Skin Disease Hospital between 2021 and 2022. The treatment for patients with psoriasis includes acitretin, methotrexate, narrow-band ultraviolet/benvitimod, and biologics. Data were collected using a structured questionnaire, physical examination, and disease severity estimation at baseline, week four, and week eight. The achievement of a ≥75% reduction in psoriasis area and severity index (PASI_75_) score from baseline to week 8 was set as the primary outcome for treatment efficacy estimation. Data were analyzed using SAS 9.4.

**RESULTS:**

A total of 560 patients with psoriasis were enrolled in this study, who were predominantly males (72.9%). The average age of patients was 48.4 years, and 38.8% of them were current smokers, 5.0% of them were former smokers. The median score of PASI among patients changed from 11.1 (interquartile range, IQR: 7.9–16.6) at baseline to 6.2 at week 4 and 3.1 at week 8, and 13.8% and 47.3% of patients with psoriasis achieved PASI_75_ at weeks 4 and 8, respectively. Logistic regression indicated that patients without tobacco smoking had a higher proportion of PASI_75_ achievement at week 8. The adjusted odds ratio (AOR) was 11.43 (95% CI: 6.91–18.89), 14.14 (95% CI: 8.27–24.20), and 3.05 (95% CI: 1.20–7.76) for non-smokers compared with smokers, current smokers, and former smokers, respectively. Moreover, former smokers had higher PASI_75_ achievement than current smokers (AOR=3.37), and patients with younger smoking initiation age, longer smoking duration, and higher smoking intensity had lower PASI_75_ achievement.

**CONCLUSIONS:**

Tobacco smoking was negatively associated with PASI_75_ achievement both in current and former smokers, and former smokers had higher PASI_75_ achievement than current smokers. The implementation of tobacco control measures is beneficial for improving treatment responses.

## INTRODUCTION

Psoriasis is a common chronic systemic inflammatory disease accompanied by severe skin damage, joint symptoms, and various comorbidities^1,2^. Globally, psoriasis affects 0.5–11.4% of the adult population, and over 120 million people suffer from it^3^. Previous studies have indicated a relatively lower prevalence of psoriasis in China, but it has increased gradually in recent years, reaching 0.47% in 2012, and there are approximately 6 million patients with psoriasis in China^4,5^. The clinical features of psoriasis are mainly erythema and silvery scale, and psoriasis can occur all over the body, with the scalp and extensor limbs being more common, usually accompanied by multifaceted comorbidities^6^. Psoriasis adversely affects patients’ quality of life and work efficiency and is treated as an important public health issue worldwide because of its increased prevalence, long disease duration, recurrence, and serious disease burden^7,8^.

Previous studies have indicated that many factors play a role in the development of psoriasis; however, the pathogenesis of psoriasis remains unclear^9^. Currently, growing evidence indicates that obesity, throat infection with streptococcus, and environmental factors (air pollution, ultraviolet radiation, tobacco smoking, etc.) are vital triggers for psoriasis initiation^10-12^. Tobacco smoking is a modifiable risk factor for general health^13^ and has negative health impacts, resulting in increased mortality in many chronic diseases^14^. Moreover, recent data indicate that >50 types of diseases are associated with tobacco smoking, and >6 million people die from smoking each year^15,16^. Smoking can induce free-radical damage and oxidative stress, increase immune cell activation, and interact with key signaling pathways in psoriasis pathogenesis^17^. As one of the key environmental pathogenic factors, tobacco smoking has proven to be an independent risk factor for the onset and development of psoriasis^18^.

In recent years, many effective therapies for psoriasis have been developed in clinical practice. For patients with mild psoriasis, treatment options include vitamin D analogs, calcineurin inhibitors, targeted phototherapy, keratolytics, and topical corticosteroids^19^. For patients with moderate to severe psoriasis, systemic treatments, including biological agents, oral agents, and phototherapy, are the mainstay treatments^20,21^. In clinical practice, clinicians widely use the psoriasis area and severity index (PASI) score to assess psoriasis severity and treat the decreased level of the PASI score as the core goal for psoriasis treatment and as a better indicator of therapeutic response, including PASI_75_, PASI_90_, and PASI_100_^22,23^. Currently, four classes of biologics (TNF inhibitors, IL-12/23 inhibitors, IL-17 inhibitors, and IL-23 inhibitors) represent one of the most significant therapeutic advancements in the field of dermatology, which have achieved satisfactory treatment effects (PASI_75_, PASI_90_, and even PASI_100_) among psoriasis patients^24^. However, psoriasis remains an incurable disease, and many patients require long-term treatment during their lifetime^2^.

As mentioned previously, the pathogenesis of psoriasis is complex and not fully elucidated^25^. Genetic factors play a critical role in the development of psoriasis, while environmental factors can exacerbate psoriasis^26^. Because some patients cannot achieve satisfactory effects even with biological agents, and many patients encounter psoriasis recurrence^2^, we suppose that environmental factors, especially tobacco smoking, might play a role during the treatment process. However, evidence of the association between tobacco smoking and psoriasis treatment effects, is still limited^27^. In this study, we aimed to understand how tobacco smoking influences the achievement of PASI_75_ after eight weeks of treatment, based on a cohort of psoriasis patients in Shanghai, China.

## METHODS

### Study population

This observational study was based on a cohort of patients with psoriasis established at the Shanghai Skin Diseases Hospital from 2021 to 2022. Zheng et al.^28^ reported that the prevalence of tobacco smoking in Shanghai was 31%. In this study, psoriasis was confirmed according to the guidelines of the Chinese Clinical Dermatology, which is in line with the global guidelines for psoriasis diagnosis and treatment. Both male and female patients with moderate-to-severe psoriasis (body surface area, BSA ≥5 and PASI ≥5), aged ≥18 years, in the inpatient department, were included, and patients with neurological or psychiatric abnormalities or who were unable to provide informed consent were excluded. This study was reviewed and approved by the Institutional Ethical Review Board of Shanghai Skin Disease Hospital (2021–44), and was registered in the Chinese Clinical Trial Registry (ChiCTR2200066403). This study strictly adhered to the Declaration of Helsinki.

### Sample size

In this study, sample size was calculated by applying the sample size estimation formula in the observational study. Zheng et al.^28^ reported that the prevalence of tobacco smoking was 31% in Shanghai. In this study, we set the prevalence of smoking as *p*=30%, type I error *α*=0.05, permitted error *δ*=15% of *p*, with a 10% non-response rate during enrollment, with the sample size formula:

*n=μ^2^_α/2_×p(1-p)/δ^2^*

indicating that at least 548 patients with psoriasis should be enrolled, which could ensure a 0.81 power (1-*β*) in this study. Finally, 560 inpatients who provided informed consent were enrolled and analyzed in this psoriasis cohort. The information that could identify individual patients after data collection was anonymized and could not be accessed by authors.

### Data collection

In this study, each patient with psoriasis underwent a physical examination, BSA, PASI, and physician global assessment (PGA), evaluation administered by dermatologists, at their hospital visits. Dermatologists would then make a proper treatment plan for each patient and discuss it with them, and the individual treatment plan was finally confirmed and implemented through physician-patient shared decision-making. The treatment plan covered four options: acitretin group (25–50 mg daily), methotrexate group (MTX, 15–20 mg per week, with folic acid supplementation), narrow-band ultraviolet (NB-UVB)/benvitimod group (2–4 times weekly, plus benvitimod topical treatment concurrently), and biologics group (ustekinumab, risankizumab, secukinumab, etc.). Data were collected using a self-designed questionnaire administered by dermatologists after training. A previous pilot study demonstrated that the content validity coefficient of the questionnaire was 0.87, and the split-half reliability coefficient was 0.85. The questionnaire consisted of: 1) demographic features, including age, sex, marital status, education level, and body mass index (BMI) etc.; 2) lifestyle habits such as tobacco smoking, physical exercise, and sleep condition etc.; 3) information on psoriasis severity (BSA, PASI, PGA), seasonality of psoriasis aggravation, and family history of psoriasis; and 4) treatment plan and PASI score evaluation at week 4 and week 8, respectively, and the records of adverse events.

### Definitions and classifications

We define a smoker as a person who smoked at least 100 cigarettes in a lifetime, a current smoker someone who still smoked at the time of investigation, and a former smoker someone who had stopped smoking for at least three months at the time of investigation^5^. We defined smoking duration as the time interval (years) between the age at investigation and age at smoking initiation for current smokers, and the time interval between the age at smoking cessation and age at smoking initiation for former smokers and then classified them into <20 years and ≥20 years^5^. Smoking intensity was defined as the number of cigarettes smoked per day and was categorized either as <20 cigarettes or ≥20 cigarettes^5^. The age of the patients was classified as <45 years and ≥45 years. Education was recorded as completed years of schooling and categorized as 0–9 years (primary or junior high school), 10–12 years (senior high school), or >12 years (college and higher). Individual monthly income was divided into three groups: <5000, 5000–10000, and >10000 RMB (1000 Chinese Renminbi about US$140). Daily sleep time was recorded as hours of sleep in the night plus naps in the daytime, and insufficient sleep was defined as a daily sleep time of <6 hours^28^.

PASI was used to assess the severity of psoriasis lesions, both for lesion area and lesion severity. The score of PASI ranges from 0 to 72, with a higher score indicating greater severity of psoriasis. PASI_75_ is defined as patients achieving ≥75% improvement in PASI score and calculated by the formula [(PASI at baseline -PASI at week t)/PASI at baseline] × 100%^29^. The efficacy of treatment was assessed by the proportion of patients achieving PASI_75_ at week 4 and week 8, and we set PASI_75_ achievement at week 8 as the primary outcome.

### Statistical analysis

Data analysis was performed using SAS software (version 9.4; SAS Institute Inc., Cary, NC, USA). Kolmogorov-Smirnov test was used to evaluate the normal data distribution for quantitative variables. Quantitative variables with a normal distribution were expressed as means and standard deviations (SD), and Student’s t-test was used to examine for significance between variables. Quantitative variables with skewed distribution were expressed as medians and interquartile ranges (IQR), and the Mann-Whitney U test was used to test for significance between variables. Qualitative variables were expressed as frequencies and percentages (%), and statistical significance between groups was tested by the chi-squared test. We applied the logistic regression to calculate the odds ratios (OR) and 95% confidence intervals (CI) to explore the association between tobacco smoking and the achievement of PASI_75_ at weeks 4 and 8 among psoriasis patients, either with current or former tobacco smoking. The generalized linear model (GLM) was used to show the trend of PASI score change from baseline to weeks 4 and week 8. Sensitivity analysis was performed to depict the association between the achievement of PASI_75_ at week 8 and smoking initiation, smoking intensity, and smoking duration among patients with psoriasis who smoked. Subgroup analysis was performed among patients with the four aforementioned treatment plans. We set statistical significance at p<0.05 (two-tailed).

## RESULTS

### Demographic and clinical features of patients with psoriasis

The age of 560 patients with psoriasis ranged 18–87 years, with an average age of 48.4 years, and 72.9% of them were male. The proportion of patients with psoriasis aged ≥45 years was 54.8%, and 81.8% of them were married; 47.7% of patients with psoriasis had college or higher education level, 22.5% had an individual monthly income >10000 RMB, and 53.9% had non-communicable diseases (NCD) comorbidities. Five hundred sixty patients with psoriasis included 217 current smokers (38.8%), 28 former smokers (5.0%), and 315 non-smokers (56.2%), and 4.6% had daily sleep time <6 hours. The median disease duration was 10.8 months (IQR: 2.3–20.0), and the median values of the BSA, PASI, and PGA scores were 15.0 (IQR: 10.0–28.0), 11.1 (IQR: 7.9–16.6) and 2.7 (IQR: 2.0–3.0), respectively. The proportions of patients with psoriasis in the acitretin, MTX, NB-UVB/benvitimod, and biologics groups were 13.2%, 19.3%, 21.6%, and 45.9%, respectively. Table 1 indicates that male patients with psoriasis had more NCD comorbidities, higher PASI and PGA scores, and higher prevalence of tobacco smoking than female patients, and the differences were all statistically significant (p<0.05).

**Table 1:** The sociodemographic and clinical characteristics, and treatment plan, among psoriasis patients in Shanghai, China (N=560)

*Characteristics*	*Total (N=560) n (%)*	*Male (N=408) n (%)*	*Female (N=152) n (%)*	*χ^2^*	p*
**Age** (years)				0.06	0.80
<45	253 (45.2)	183 (44.9)	70 (46.1)		
≥45	307 (54.8)	225 (55.1)	82 (53.9)		
**Education level**				3.85	0.15
Junior high and lower	159 (28.4)	110 (27.0)	49 (32.2)		
Senior high	134 (23.9)	106 (26.0)	28 (18.4)		
College and higher	267 (47.7)	192 (47.0)	75 (49.3)		
**Marital status**				0.03	0.87
Unmarried	102 (18.2)	75 (18.4)	27 (17.8)		
Married	458 (81.8)	333 (81.6)	125 (82.2)		
**Individual monthly income** (RMB)				1.48	0.48
<5000	214 (38.2)	152 (37.2)	62 (40.8)		
5000–10000	220 (39.3)	159 (39.0)	61 (40.1)		
>10000	126 (22.5)	97 (23.8)	29 (19.1)		
**Residency status**				1.58	0.21
Local resident	337 (60.2)	252 (61.8)	85 (55.9)		
Non-local resident	223 (39.8)	156 (38.2)	67 (44.1)		
**Non-communicable diseases** (NCD)				14.47	<0.01
Yes	302 (53.9)	240 (58.8)	62 (40.8)		
No	258 (46.1)	168 (41.2)	90 (59.2)		
**Tobacco smoking status**				82.92	<0.01
Current smoker	217 (38.8)	199 (48.8)	18 (11.8)		
Former smoker	28 (5.00)	27 (6.6)	1 (0.7)		
Non-smoker	315 (56.2)	182 (44.6)	133 (87.5)		
**Total daily sleep time** (hours)				0.23	0.63
<6	26 (4.6)	20 (4.9)	6 (4.0)		
≥6	534 (95.4)	388 (95.1)	146 (96.0)		
**Disease duration of psoriasis,** median (IQR)	10.8 (2.3-20.0)	10.3 (2.1–19.7)	12.3 (2.9–21.9)	2.17	0.14
BSA score before treatment, median (IQR)	15.0 (10.0–28.0)	16.0 (11.0–29.0)	13.2 (8.5–23.7)	3.26	0.07
PASI score before treatment, median (IQR)	11.1 (7.9–16.6)	12.0 (8.4–18.0)	9.6 (6.8–13.8)	20.03	<0.01
PGA score before treatment, median (IQR)	2.7 (2.0–3.0)	2.7 (2.3–3.0)	2.3 (2.0–3.0)	18.14	<0.01
**Treatment plan for patients**				18.9	<0.01
Acitretin	74 (13.2)	62 (15.2)	12 (7.9)		
Methotrexate (MTX)	108 (19.3)	70 (17.2)	38 (25.0)		
Narrow-band ultraviolet (NB-UVB)/benvitimod	121 (21.6)	75 (18.4)	46 (30.3)		
Biologics (ustekinumab, risankizumab, etc.)	257 (45.9)	201 (49.3)	56 (36.8)		

RMB: 1000 Chinese Renminbi about US$140. BSA: body surface area. PASI: psoriasis area and severity index. PGA: physician global assessment.

* Chi-squared test was used to derive the p value for the comparison between male and female patients. IQR: interquartile range.

### Demographic features of patients with different tobacco smoking conditions

Data in Table 2 give the demographic and dermatological features of psoriasis patients with different tobacco smoking status. In comparison with non-smokers, patients with tobacco smoking habit had a lower proportion of age <45 years and a lower proportion of college and higher education level, were predominately males, and had more NCD comorbidities; the differences were all statistically significant (p<0.05). Differences in age, sex, education level, and NCD comorbidities were identified between current smokers and non-smokers, as well as between former smokers and non-smokers (Table 2).

**Table 2:** Sociodemographic characteristics, smoking initiation, intensity, and duration, among psoriasis patients in Shanghai, China (N=560)

*Characteristics*	*Tobacco smoking status*			
	*Smoker (N=245) n (%)*	*Current smoker (N=217) n (%)*	*Former smoker (N=28) n (%)*	*Non-smoker (N=315) n (%)*
**Age** (years) <45 years^a-d^	89 (36.3)	83 (38.3)	6 (21.4)	164 (52.1)
**Male patients^a-d^**	226 (92.2)	199 (91.7)	27 (96.4)	182 (57.8)
**Married patients^d^**	208 (84.9)	185 (85.3)	23 (82.1)	250 (79.4)
**Education level^a-d^**				
Junior high and lower	78 (31.8)	65 (30.0)	13 (46.4)	81 (25.7)
Senior high	77 (31.4)	69 (31.8)	8 (28.6)	57 (18.1)
College and higher	90 (36.7)	83 (38.2)	7 (25.0)	177 (56.2)
**Patients with NCD^a,b,d^**	151 (61.6)	134 (61.8)	17 (60.7)	151 (47.9)
**Daily sleep time <6 hours/day^d^**	13 (5.3)	10 (4.6)	3 (10.7)	13 (4.1)
**Diseases duration of psoriasis** (months)^e^, median (IQR)	10.7 (2.1–19.7)	10.1 (2.0–19.1)	14.3 (10.8–22.0)	10.8 (2.6–20.9)
**Scores before treatment**				
BSA^e^, median (IQR)	15.0 (10.0–27.0)	15.0 (10.0–28.0)	15.0 (13.0–25.8)	15.5 (10.0–29.0)
PASI^e^, median (IQR)	11.2 (8.0–17.7)	11.2 (8.0–18.6)	10.8 (9.0–16.9)	11.0 (7.8–15.6)
PGA^e^, median (IQR)	2.7 (2.0–3.0)	2.7 (2.0–3.0)	2.7 (2.0–3.0)	2.7 (2.0–3.0)
**Treatment plan for patients^d^**				
Acitretin	37 (15.1)	34 (15.7)	3 (10.7)	37 (11.8)
Methotrexate	45 (18.4)	39 (18.0)	6 (21.4)	63 (20.0)
Narrow-band ultraviolet/benvitimod	51 (20.8)	46 (21.2)	5 (17.9)	70 (22.2)
Biologics (ustekinumab, risankizumab, etc)	112 (45.7)	98 (45.1)	14 (50.0)	145 (46.0)
**Age of smoking initiation** (years)^e^, median (IQR)	23.0 (20.0–27.0)	23.0 (20.0–27.0)	25.0 (20.0–30.0)	-
**Years of tobacco smoking^e^,** median (IQR)	26.0 (15.0–40.0)	25.0 (15.0–40.0)	31.5 (14.5–43.0)	-
**Smoking duration <20 years^d^**	78 (31.8)	69 (31.8)	9 (32.1)	-
**Daily consumed cigarettes^e^,** median (IQR)	20.0 (10.0–20.0)	20.0 (10.0–20.0)	12.5 (10.0–20.0)	-
**Smoking intensity <20 cigarettes/day^d^**	115 (46.9)	98 (45.2)	17 (60.7)	-

BSA: body surface area. PASI: psoriasis area and severity index. PGA: physician global assessment.

a The differences between smoker and non-smoker was statistically significant (p<0.05).

b The differences between current smoker and non-smoker was statistically significant (p<0.05).

c The differences between former smoker and non-smoker was statistically significant (p<0.05).

d The chi-squared test was used to derive the p value for the comparison between groups.

e Mann-Whitney U tests was used to derive the p value for the comparison between groups. IQR: interquartile range.

Patients with psoriasis who smoked tobacco tended to have higher PASI scores, but the disease duration, BSA score, PGA score, and treatment plan were similar to those of patients who did not smoke. In all 245 psoriasis patients with tobacco smoking, the median values of smoking initiation age, tobacco smoking years, and daily consumed cigarettes were 23.0 (IQR: 20.0–27.0), 26.0 (IQR: 15.0–40.0) and 20.0 (IQR: 10.0–20.0), respectively. Current smokers had a younger smoking initiation age, had a lower proportion of smoking duration <20 years, and smoking intensity <20 cigarettes/day than former smokers (Table 2).

### PASI_75_ achievement at week 4 and week 8 among patients with psoriasis

**Figure 1:**
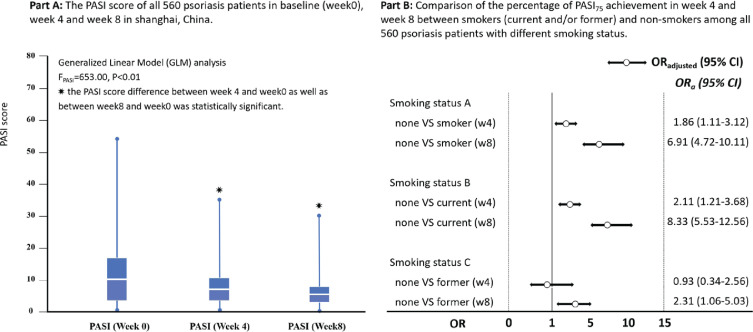
The psoriasis area and severity index (PASI) score evaluation among psoriasis patients at baseline, week 4 and week 8, and comparison of the percentage of PASI_75_ achievement from baseline to week 4 and week 8 among patients with different tobacco smoking status in Shanghai, China (N=560)

The median score of PASI among patients with psoriasis changed gradually from 11.1 (IQR: 7.9–16.6) at baseline to 6.2 (IQR: 3.4–10.3) at week 4, and 3.1 (IQR: 1.2–6.3) at week 8 after treatment, GLM analysis indicated a statistical significance for PASI score change (F=243.5, p<0.01). Among the 560 patients with psoriasis, 13.8% (77/560) and 47.3% (265/560) achieved PASI_75_ at weeks 4 and 8, respectively. Figure 1 indicates that patients with psoriasis who did not smoke had higher PASI_75_ achievement in week 4 (OR=1.86; 95% CI: 1.11–3.12) and week 8 (OR=6.91; 95% CI: 4.72–10.11) than those who smoked; the findings were consistent in week 4 and week 8 for non-smokers compared with current smokers, but was only in week 8 for non-smokers compared to former smokers (OR=2.31; 95% CI: 1.06–5.03) (Figure 1).

### Association between tobacco smoking and PASI_75_ achievement in week 8

Patients with psoriasis who smoked tobacco had a lower PASI_75_ achievement in week 8 than those without tobacco smoking (p<0.05). Female patients, patients with an education level of college and higher, without NCD comorbidities, with daily sleep time ≥6 hours, and with biologics treatment, tended to have a higher PASI_75_ achievement at week 8; the differences were statistically significant (p<0.05). Multivariable logistic regression analysis with potential confounders adjustment (age, sex, education level, NCD, daily sleep time, and treatment plan) indicated that patients without tobacco smoking had a higher proportion of PASI_75_ achievement at week 8, the adjusted odds ratio (AOR) was 11.43 (95% CI: 6.91–18.89), 14.14 (95% CI: 8.27–24.20), and 3.05 (95% CI: 1.20–7.76), for non-smokers compared with smokers, current smokers and former smokers, respectively (Table 3).

**Table 3:** Factors associated with the achievement of PASI_75_ after 8 weeks of treatment among psoriasis patients in Shanghai, China (N=560)

*Variables*	*PASI_75_ in Week 8 n (%)*	*Model A*		*Model B*		*Model C*	
		*OR*	*95% CI*	*OR*	*95% CI*	*OR*	*95 % CI*
**Smoking status A^a^**							
Smoker ®	55 (22.5)	1		-	-	-	-
Non-smoker	210 (66.7)	**11.43**	**6.91–18.89**	-	-	-	-
**Smoking status B^a^**							
Current smoker ®	42 (19.4)	-	-	1		-	-
Non-smoker	210 (66.7)	-	-	**14.14**	**8.27–24.20**	-	-
**Smoking status C^a^**							
Former smoker ®	13 (46.4)	-	-	-	-	1	
Non-smoker	210 (66.7)	-	-	-	-	**3.05**	**1.20–7.76**
**Age** (years)^a^							
<45	131 (54.8)	1.52	0.93–2.49	1.32	0.79–2.19	1.50	0.79–2.83
≥45 ®	134 (46.7)	1		1		1	
**Sex^a^**							
Male	177 (43.4)	0.84	0.51–1.39	0.89	0.54–1.49	1.10	0.72–3.36
Female ®	88 (57.9)	1		1		1	
**Education level^a^**							
Junior high and lower ®	60 (37.7)	1		1		1	
Senior high	61 (45.5)	1.61	0.89–2.90	**1.99**	**1.06–3.72**	1.56	0.72–3.36
College and higher	144 (53.9)	1.30	0.74–2.27	1.38	0.77–2.47	1.30	0.65–2.62
**NCD^a^**							
Yes ®	128 (42.4)	1		1		1	
No	137 (53.1)	1.32	0.86–2.03	1.29	0.82–2.03	1.14	0.67–1.97
**Daily sleep time** (hours)^a^							
<6 ®	7 (26.9)	1		1		1	
≥6	258 (48.3)	1.87	0.70–4.99	2.52	0.83–7.66	2.81	0.89–8.83
**Treatment plan^a^**							
Acitretin ®	14 (18.9)	1		1		1	
Methotrexate	37 (34.3)	**2.37**	**1.08–5.19**	**2.31**	**1.04–5.14**	1.83	0.80–4.22
NB-UVB/benvitimod	46 (38.0)	**2.98**	**1.36–6.54**	**2.59**	**1.16–5.78**	2.26	0.97–5.25
Biologics	168 (65.4)	**13.37**	**6.38–28.02**	**12.59**	**5.89–26.91**	**13.05**	**5.72–29.74**

a The differences of PASI_75_ achievement in week 8 between groups was statistically significant (p<0.05). Model A: Multivariable logistic regression to explore association between tobacco smoking and the achievement of PASI_75_ in week 8 among smokers (N=245) and non-smokers (N=315). Model B: Multivariable logistic regression to explore association between tobacco smoking and the achievement of PASI_75_ in week 8 among current smokers (N=217) and non-smokers (N=315). Model C: Multivariable logistic regression to explore association between tobacco smoking and the achievement of PASI_75_ in week 8 among former smokers (N=28) and non-smokers (N=315). NCD: non-communicable diseases. NB-UVB: Narrow-band ultraviolet. ® Reference categories.

### Subgroup and sensitivity analysis

**Figure 2:**
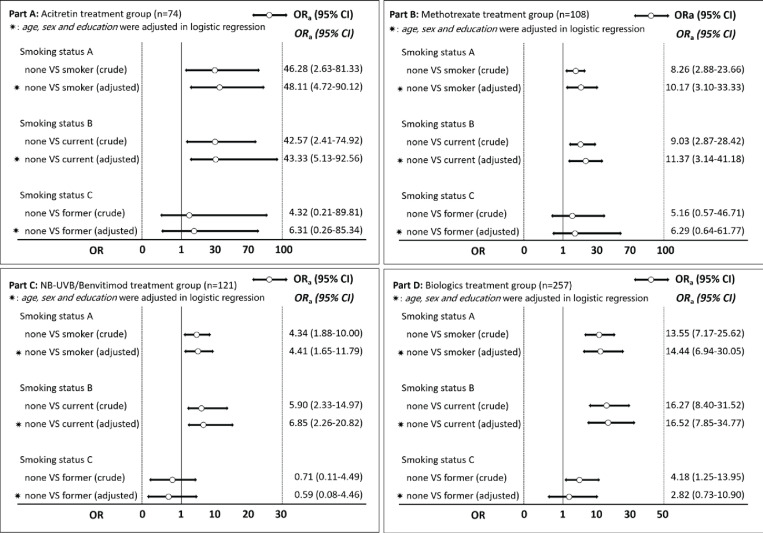
The comparison of PASI_75_ achievement from baseline to week 8 among patients with different tobacco smoking status based on the logistic regression model and in different treatment groups (Acitretin, MTX, NB-UVB/benvitimod and biologics) in Shanghai, China (N=560)

Subgroup analysis was performed to demonstrate the association between tobacco smoking and PASI_75_ achievement at week 8 for patients with different treatment plans. Figure 2 indicates that smokers had lower PASI_75_ achievement at week 8 than non-smokers in the acitretin, MTX, NB-UVB/benvitimod, and biologics groups, especially among current smokers. Current smokers tended to have lower PASI_75_ achievement at week 8 than non-smokers, but the difference was not statistically significant (p>0.05).

We also explored the association between PASI_75_ achievement at week 8 and tobacco smoking habits among 245 patients with psoriasis who smoked tobacco. The data in Table 4 show that former smokers had higher PASI_75_ achievement at week 8 than non-smokers (OR=3.61; 95% CI: 1.60–8.16), even after adjusting for potential confounders (AOR=3.37; 95% CI: 1.40–8.10). The findings also showed that psoriasis patients with a younger smoking initiation age, longer smoking duration, and higher smoking intensity had lower PASI_75_ achievement at week 8, but the difference was not statistically significant (p>0.05).

**Table 4:** The association between the achievement of PASI_75_ after 8 weeks of treatment and smoking initiation age, smoking intensity and smoking duration among psoriasis patients with smoking in Shanghai, China (N=245)

*Variables*	*PASI_75_ in Week 8 n (%)*	*Model 1*		*Model 2*	
		*OR*	*95% CI*	*AOR*	*95 % CI*
**Tobacco smoking status**					
Current ®	42 (19.4)	1		1	
Former	13 (46.4)	3.61	1.60–8.16	3.37	1.40–8.10
**Smoking initiation age** (years)					
<18 ®	4 (15.4)	1		1	
18–25	28 (23.9)	1.73	0.55–5.45	1.72	0.52–5.66
>25	23 (22.6)	1.60	0.50–5.12	1.25	0.36–4.26
**Smoking duration** ( years )					
<20 ®	18 (23.1)	1		1	
≥20	37 (22.2)	0.95	0.50–1.80	0.52	0.17–1.66
**Smoking intensity** (cigarettes/day)					
<20 ®	27 (23.5)	1		1	
≥20	28 (21.5)	0.90	0.49–1.63	0.85	0.44–1.61

Model 1: Univariable logistic regression to explore association between tobacco smoking and the achievement of PASI_75_ in week 8 among psoriasis patients with smoking habit. Model 2: Multivariable logistic regression to explore association between tobacco smoking and the achievement of PASI_75_ in week 8 among psoriasis patients with smoking habit, with the adjustment of age, sex, and education level. AOR: adjusted odds ratio. PASI: psoriasis area and severity index. ® Reference categories.

## DISCUSSION

This study recruited 560 patients with psoriasis in Shanghai to explore the influence of tobacco smoking on the efficacy of systemic treatments based on real-world clinical data. This study showed that tobacco smoking was negatively associated with PASI_75_ achievement, both for patients with current and former tobacco smoking habits. Moreover, former smokers had higher PASI_75_ achievement than current smokers, and patients with a younger smoking initiation age, longer smoking duration, and higher smoking intensity had lower PASI_75_ achievement at week 8.

In practice, the decreased PASI score was treated as the core goal for psoriasis treatment, and the levels of PASI_75_, PASI_90,_ and PASI_100_ were viewed as better indicators of therapeutic response than the absolute decreased value of PASI score^22,23^. In this study, the overall PASI_75_ achievement was 13.8% at week 4 and 47.3% at week 8, which is consistent with previous studies^29-31^. Griffiths et al.^29^ reported a <20% improvement of at least 75% in the PASI score among patients with psoriasis receiving etanercept or ustekinumab treatment at week 4, and approximately 40% and 58% of PASI_75_ achievement for etanercept and ustekinumab treatment at week 8, respectively. In a randomized clinical trial by Gordon et al.^30^, adalimumab treatment targeted for TNF_α_ neutralization achieved <80% PASI reduction on average at week 8, and approximately 60% of patients with psoriasis achieved PASI_75_ at week 8 with infliximab treatment, which was reported by Kristian et al.^31^. The American Academy of Dermatology National Psoriasis Foundation guidelines recommend the concurrent use of biologics, oral agents, and phototherapy for patients with moderate-to-severe psoriasis^26^. In this study, patients treated with biologics achieved better efficacy (65.4% achieved PASI_75_ at week 8) than those treated with acitretin, methotrexate, or NB-UVB. Previous studies have reported that immunosuppressive agents such as methotrexate and acitretin are effective in the treatment of psoriasis, and biologics that selectively block the steps in the inflammatory cascade provide additional therapies for psoriasis and have better treatment efficacy^32^.

Tobacco smoking is a well-established modifiable risk factor^13^ and is positively associated with the initiation and severity of psoriasis^18^. In this study, we noticed that tobacco smoking was negatively associated with the PASI_75_ achievement at week 8 during systemic treatment. Patients without tobacco smoking had a 14.14 (95% CI: 8.27–24.20) and 3.05 (95% CI: 1.20–7.76) times higher chance of achieving PASI_75_ at week 8 than patients with current or former tobacco smoking, respectively. Moreover, this study also indicated that patients with younger smoking initiation age, longer smoking duration, and higher smoking intensity had lower PASI_75_ achievement at week 8; the findings were in line with previous studies. Zhou et al.^33^ reported that psoriasis disease improvement at six months in former smokers was poorer than in non-smokers (OR=0.80; 95% CI: 0.67–0.95) in a meta-analysis. Gupta et al.^34^ found a lower percentage of tobacco smokers achieving PASI_75_ at three months compared to conventional drug therapy for plaque psoriasis. Previous studies have indicated that excessive activation of the adaptive immune system is the kernel of psoriasis pathogenesis, and a variety of cell types, including keratinocytes, natural killer T cells, and macrophages, secrete cytokines that activate myeloid dendritic cells and play a role in the initiation and development of psoriasis^25^. Tobacco smoking may trigger the initiation and development of psoriasis through oxidative, inflammatory, and genetic mechanisms. Nicotine also stimulates innate immune cells, and these cells release cytokines that activate T lymphocytes and perpetuate a cycle of chronic inflammation^17^, which is integral to the pathogenesis of psoriasis and might contribute to the lower treatment efficacy than in patients with a smoking habit.

Smoking cessation is the most powerful and cost-effective intervention available in clinical settings for the prevention of disease, disability, and death^5^, but currently, studies assessing the impact of smoking cessation on psoriasis are limited^33^. In this study, former smokers had a significantly weaker association between smoking and PASI_75_ achievement than current smokers (AOR=3.05 and 14.14) compared with non-smokers. In subgroup analysis among psoriasis patients with tobacco smoking, former smokers had higher PASI_75_ achievement at week 8 than current smokers (OR=3.61), even after adjusting for potential confounders (AOR=3.37). Zhou et al.^33^ reported that smoking cessation benefits patients with psoriasis under treatment, based on a pooled analysis. Therefore, implementing tobacco control measures among patients with psoriasis is beneficial for improving the treatment response. Moreover, illness can be a powerful motivation to quit. The provision of cessation guides and bedside support and delivery of effective patient-centered, culturally sensitive tobacco cessation services can forestall the discomfort of nicotine withdrawal and permit the chance of successful quitting.

Previous studies have indicated that tobacco smoking is associated with the initiation and severity of psoriasis^5,18^. In this study, patients with psoriasis and tobacco smoking had a higher median score of PASI than those without tobacco smoking, but the difference was not statistically significant. This might be due to selection bias because psoriasis patients enrolled in this study were all patients with moderate to severe psoriasis, who tended to have higher scores of BSA and PASI, even without tobacco smoking, which diminished the association between tobacco smoking and psoriasis severity. We also observed in the subgroup analysis that patients with psoriasis who smoked tobacco had a lower proportion of PASI_75_ achievement at week 8, and the findings were consistent regardless of their treatment plan. However, the influence of tobacco smoking on PASI_75_ achievement among patients with psoriasis was stronger in the acitretin treatment group (OR=48.11) than in the MTX (OR=10.17), NB-UVB (OR=4.41) and biologics (OR=14.44) treatment groups, this might be due to the small sample size in the acitretin treatment group; however, the true reason for these differences should be explored in future studies.

### Strengths and limitations

A key strength of this study was the longitudinal design for data collection among 560 enrolled patients during their treatment based on real-world clinical circumstances, and the eight weeks of follow-up time ensured 100% compliance among patients with psoriasis. Moreover, the clinical data of patients with psoriasis were extracted from the Health Information System (HIS) directly without recall bias, which resulted in high data quality, which is another strength of this study.

This study has some limitations. First, all 560 patients were enrolled in one hospital, which ensured high internal validity; however, the generalization of the findings to patients with psoriasis in China or even in Shanghai was limited. Second, tobacco smoking status among patients with psoriasis is self-reported, which might lead to recall bias, and the missing information on secondhand smoke exposure might underestimate the influence of tobacco smoking on treatment efficacy to some degree. The self-designed questionnaire for data collection might also lead to some information bias. Third, the time points for treatment efficacy assessment were set at weeks 4 and 8, with week 8 as the primary outcome in this study. Week 12 is more commonly used for efficacy assessment; therefore, the rate of PASI_75_ achievement at week 8 in this study had limited comparability with other studies, so the incorporation of more assessment time points should be considered. Fourth, only tobacco smoking habits were assessed in this study, and the influence and interaction of other lifestyle habits, including alcohol consumption, lack of physical exercise, and high-fat diet and beverage consumption, should be considered in future studies^35,36^. Fifth, we applied a logistic regression model in this study to explore the association between tobacco smoking and the achievement of PASI_75_ among patients with psoriasis. Considering the longitudinal measurement of PASI, mixed models would be more appropriate to show the association and the interaction, which should be used in future studies. Finally, the quality-of-life assessment is another indicator of treatment efficacy but was not included in this study; therefore, the Dermatology Life Quality Index (DLQI), Short-Form-36 (SF-36), and Dermatology Quality of Life Scale (DQOLS) should be incorporated in future studies.

## CONCLUSIONS

Tobacco smoking was negatively associated with the proportion of PASI_75_ achievement among patients with psoriasis, both for those with current or former tobacco smoking habits. Moreover, former smokers had a higher proportion of PASI_75_ achievement than current smokers. We recommend the implementation of tobacco control measures, the provision of patient-centered, culturally sensitive cessation guides, and bedside support to improve the treatment response among patients with psoriasis.

## Data Availability

The data supporting this research are available from the authors on reasonable request. The request should state the title and aim of the research for which the data are requested.

## References

[1] Griffiths CEM, Armstrong AW, Gudjonsson JE, Barker JNWN. Psoriasis. Lancet. 2021;397(10281):1301-1315. doi:10.1016/S0140-6736(20)32549-6 33812489

[2] Park SY, Kim KH. What factors influence on dermatology-related life quality of psoriasis patients in South Korea? Int J Environ Res Public Health. 2021;18(7):3624. doi:10.3390/ijerph18073624 33807295 PMC8038132

[3] Michalek IM, Loring B, John SM. A systematic review of worldwide epidemiology of psoriasis. J Eur Acad Dermatol Venereol. 2017;31(2):205-212. doi:10.1111/jdv.13854 27573025

[4] Ding X, Wang T, Shen Y, et al. Prevalence of psoriasis in China: a population-based study in six cities. Eur J Dermatol. 2012;22(5):663-667. doi:10.1684/ejd.2012.1802 22910173

[5] Wei L, Chen S, Zhang Z, et al. Prevalence of tobacco smoking and its association with disease severity among patients with psoriasis in China: a cross-sectional study. Front Med (Lausanne). 2022;9:883458. doi:10.3389/fmed.2022.883458 35646971 PMC9133951

[6] Korman NJ. Management of psoriasis as a systemic disease: what is the evidence? Br J Dermatol. 2020;182(4):840-848. doi:10.1111/bjd.18245 31225638 PMC7187293

[7] Michelsen B, Uhlig T, Sexton J, et al. Health-related quality of life in patients with psoriatic and rheumatoid arthritis: data from the prospective multicentre NOR-DMARD study compared with Norwegian general population controls. Ann Rheum Dis. 2018;77(9):1290-1294. doi:10.1136/annrheumdis-2018-213286 29875096

[8] Pilon D, Teeple A, Zhdanava M, et al. The economic burden of psoriasis with high comorbidity among privately insured patients in the United States. J Med Econ. 2019;22(2):196-203. doi:10.1080/13696998.2018.1557201 30523738

[9] Armstrong AW, Read C. Pathophysiology, clinical presentation, and treatment of psoriasis: a review. JAMA. 2020;323(19):1945-1960. doi:10.1001/jama.2020.4006 32427307

[10] Kobayashi K, Kamekura R, Kato J, et al. Cigarette smoke underlies the pathogenesis of palmoplantar pustulosis via an IL-17A-induced production of IL-36γ in tonsillar epithelial cells. J Invest Dermatol. 2021;141(6):1533-1541.e4. doi:10.1016/j.jid.2020.09.028 33188781

[11] Pezzolo E, Naldi L. The relationship between smoking, psoriasis and psoriatic arthritis. Expert Rev Clin Immunol. 2019;15(1):41-48. doi:10.1080/1744666X.2019.1543591 30380949

[12] Lu J, Shi Y. A review of disease burden and clinical management for generalized pustular psoriasis in China. Expert Rev Clin Immunol. 2022;18(10):1023-1032. doi:10.1080/1744666X.2022.2118716 36040447

[13] Lee VWY, Li A, Li JTS. Burden of smoking in Asia-Pacific countries. Tob Induc Dis. 2021;19(April):28. doi:10.18332/tid/133633 33867905 PMC8049108

[14] Forey BA, Thornton AJ, Lee PN. Systematic review with meta-analysis of the epidemiological evidence relating smoking to COPD, chronic bronchitis and emphysema. BMC Pulm Med. 2011;11:36. doi:10.1186/1471-2466-11-36 21672193 PMC3128042

[15] Gandini S, Botteri E, Iodice S, et al. Tobacco smoking and cancer: a meta-analysis. Int J Cancer. 2008;122(1):155-164. doi:10.1002/ijc.23033 17893872

[16] Carter BD, Abnet CC, Feskanich D, et al. Smoking and mortality--beyond established causes. N Engl J Med. 2015;372(7):631-640. doi:10.1056/NEJMsa1407211 25671255

[17] Lipa K, Zając N, Owczarek W, Ciechanowicz P, Szymańska E, Walecka I. Does smoking affect your skin? Postepy Dermatol Alergol. 2021;38(3):371-376. doi:10.5114/ada.2021.103000 34377115 PMC8330869

[18] Salihbegovic EM, Kurtalic N, Omerkic E. Smoking cigarettes and consuming alcohol in patients with psoriasis. Mater Sociomed. 2021;33(1):30-33. doi:10.5455/msm.2021.33.30-33 34012347 PMC8116091

[19] Soleymani T, Hung T, Soung J. The role of vitamin D in psoriasis: a review. Int J Dermatol. 2015;54(4):383-392. doi:10.1111/ijd.12790 25601579

[20] Gottlieb AB, Blauvelt A, Thaçi D, Leonardi CL, Poulin Y, Drew J, Peterson L, Arendt C, Burge D, Reich K. Certolizumab pegol for the treatment of chronic plaque psoriasis: Results through 48 weeks from 2 phase 3, multicenter, randomized, double-blinded, placebo-controlled studies (CIMPASI-1 and CIMPASI-2). J Am Acad Dermatol. 2018;79(2):302-314. doi:10.1016/j.jaad.2018.04.012 29660421

[21] Bagel J, Duffin KC, Moore A, et al. The effect of secukinumab on moderate-to-severe scalp psoriasis: Results of a 24-week, randomized, double-blind, placebo-controlled phase 3b study. J Am Acad Dermatol. 2017;77(4):667-674. doi:10.1016/j.jaad.2017.05.033 28780364

[22] Armstrong AW, Gooderham M, Warren RB, et al. Deucravacitinib versus placebo and apremilast in moderate to severe plaque psoriasis: Efficacy and safety results from the 52-week, randomized, double-blinded, placebo-controlled phase 3 POETYK PSO-1 trial. J Am Acad Dermatol. 2023;88(1):29-39. doi:10.1016/j.jaad.2022.07.002 35820547

[23] Kiltz U, Sfikakis PP, Gaffney K, et al. Secukinumab use in patients with moderate to severe psoriasis, psoriatic arthritis and ankylosing spondylitis in real-world setting in Europe: baseline data from SERENA Study. Adv Ther. 2020;37(6):2865-2883. doi:10.1007/s12325-020-01352-8 32378070 PMC7467439

[24] Papp KA, Langley RG, Lebwohl M, et al; PHOENIX 2 study investigators. Efficacy and safety of ustekinumab, a human interleukin-12/23 monoclonal antibody, in patients with psoriasis: 52-week results from a randomised, double-blind, placebo-controlled trial (PHOENIX 2). Lancet. 2008;371(9625):1675-1684. doi:10.1016/S0140-6736(08)60726-6 18486740

[25] Nestle FO, Kaplan DH, Barker J. Mechanisms of disease: psoriasis. New England Journal of Medicine. 2009;361(5):496-509. doi:10.1056/NEJMra0804595 19641206

[26] Alwan W, Nestle FO. Pathogenesis and treatment of psoriasis: exploiting pathophysiological pathways for precision medicine. Clin Exp Rheumatol. 2015;33(5 Suppl 93):S2-S6. Accessed January 30, 2024. https://www.clinexprheumatol.org/abstract.asp?a=9901 26472336

[27] Naldi L, Chatenoud L, Linder D, et al. Cigarette smoking, body mass index, and stressful life events as risk factors for psoriasis: results from an Italian case-control study. J Invest Dermatol. 2005;125(1):61-67. doi:10.1111/j.0022-202X.2005.23681.x 15982303

[28] Zheng Q, Kuai L, Jiang W, et al. Clinical feature, lifestyle behavior and non-communicable diseases comorbidities among psoriasis patients in Shanghai: gender disparity analysis based on a cross-sectional study. Clin Cosmet Investig Dermatol. 2022;15:2751-2762. doi:10.2147/CCID.S393697 PMC9762258 36545501

[29] Griffiths CE, Strober BE, van de Kerkhof P, et al; ACCEPT Study Group. Comparison of ustekinumab and etanercept for moderate-to-severe psoriasis. N Engl J Med. 2010;362(2):118-128. doi:10.1056/NEJMoa0810652 20071701

[30] Gordon KB, Langley RG, Leonardi C, et al. Clinical response to adalimumab treatment in patients with moderate to severe psoriasis: double-blind, randomized controlled trial and open-label extension study. J Am Acad Dermatol. 2006;55(4):598-606. doi:10.1016/j.jaad.2006.05.027 17010738

[31] Kristian R, Frank ON, Kim P, et al. Infliximab induction and maintenance therapy for moderate to severe psoriasis: a phase III, muticentre, double blind trial. Lancet. 2005;366:1367-1374. doi:10.1016/S0140-6736(05)67566-6 16226614

[32] Menter A, Feldman SR, Weinstein GD, et al. A randomized comparison of continuous vs. intermittent infliximab maintenance regimens over 1 year in the treatment of moderate-to-severe plaque psoriasis. J Am Acad Dermatol. 2007;56(1):31.e1-31.e15. doi:10.1016/j.jaad.2006.07.017 17097378

[33] Zhou H, Wu R, Kong Y, Zhao M, Su Y. Impact of smoking on psoriasis risk and treatment efficacy: a meta-analysis. J Int Med Res. 2020;48(11):0300060520964024. doi:10.1177/0300060520964024 33121308 PMC7780610

[34] Gupta AK, Pandey SS, Pandey BL. Effectiveness of conventional drug therapy of plaque psoriasis in the context of consensus guidelines: a prospective observational study in 150 patients. Ann Dermatol. 2013;25(2):156-162. doi:10.5021/ad.2013.25.2.156 23717005 PMC3662907

[35] Michalski P, Palazzo-Michalska V, Michalska-Bańkowska A, Bańkowski M, Grabarek BO. Impact of alcohol consumption, smoking, and diet on the severity of plaque psoriasis: a comprehensive assessment using clinical scales and quality of life measures. Med Sci Monit. 2023;29:e941255. doi:10.12659/MSM.941255 37528577 PMC10405633

[36] Michalski P, Palazzo-Michalska V, Buda P, et al. A crossroads between dietary habits, alcohol consumption, and smoking in the clinical course of psoriasis: a narrative review. Postepy Dermatol Alergol. 2023;40(5):599-605. doi:10.5114/ada.2023.129308 38028418 PMC10646720

